# An eye-tracking study on visual perception of vegetation permeability in virtual reality forest exposure

**DOI:** 10.3389/fpubh.2023.1089423

**Published:** 2023-01-24

**Authors:** Chang Li, Chunlan Du, Shutong Ge, Tong Tong

**Affiliations:** ^1^School of Architecture and Urban Planning, Suzhou University of Science and Technology, Suzhou, China; ^2^Key Laboratory of New Technology for Construction of Cities in Mountain Area, Ministry of Education, Chongqing University, Chongqing, China; ^3^School of Architecture and Urban Planning, Chongqing University, Chongqing, China

**Keywords:** vegetation permeability, eye-tracking, visual preference, interaction effect, virtual reality

## Abstract

Previous studies have confirmed the significant effects of single forest stand attributes, such as forest type (FT), understory vegetation cover (UVC), and understory vegetation height (UVH) on visitors' visual perception. However, rarely study has yet clearly determined the relationship between vegetation permeability and visual perception, while the former is formed by the interaction of multiple forest stand attributes (i.e., FT, UVC, UVH). Based on a mixed factor matrix of FT (i.e., coniferous forests and broadleaf), UVC level (i.e., 10, 60, and 100%), and UVH level (0.1, 1, and 3 m), the study creates 18 immersive virtual forest videos with different stand attributes. Virtual reality eye-tracking technology and questionnaires are used to collect visual perception data from viewing virtual forest videos. The study finds that vegetation permeability which is formed by the interaction effect of canopy density (i.e., FT) and understory density (i.e., UVC, UVH), significantly affects participant's visual perception: in terms of visual physiology characteristics, pupil size is significantly negatively correlated with vegetation permeability when participants are viewing virtual reality forest; in terms of visual psychological characteristics, the understory density formed by the interaction of UVC and UVH has a significant impact on visual attractiveness and perceived safety and the impact in which understory density is significantly negatively correlated with perceived safety. Apart from these, the study finds a significant negative correlation between average pupil diameter and perceived safety when participants are viewing virtual reality forests. The findings may be beneficial for the maintenance and management of forest parks, as well as provide insights into similar studies to explore urban public green spaces.

## 1. Introduction

Urban forest plays an important role in public health. Numerous studies in Asia, Europe, and North America have shown that forest environments provide individuals with health benefits by stimulating the senses such as vision, hearing, and smell (1–3). Therefore, forest therapy has become a preventive medicine measure to strengthen the immune system and improve mental health (4–6). In terms of physical health, exposure to forest environments can bring about cardiorespiratory fitness (2, 7), prevention of cardiovascular disease (1, 8), and anti-inflammatory benefits (9). In terms of mental health, forest environments can help promote physiological relaxation (10), reduce anxiety, depression, and stress (11–13), as well as relieve fatigue, and arouse positive emotions (14–16).

In recent years, to verify the potential benefits of forests in health promotion and disease prevention, an increasing number of studies address the visitors' experience of sit-down and walking behaviors in the forest, namely Shinrin-yoku (1–3). Most of them focus on cognitive preferences which are brought about by tourists' visual experience and investigate a wide range of forest variables, especially the physical environment features such as stand structure (10, 17), canopy density (18, 19), tree cover density (20), openness-enclosure (21, 22), vegetation density (23, 24), and species composition (25). These surveys are designed to assess the stand structure features of the forest that visitors liked and disliked through visual variables, and the results indicate that physical structural features of the forest influence visitors' visual environmental preferences (22, 26, 27). For example, natural, vibrant, and plant-rich forests are more favored than human-induced or damaged forests (22, 26, 27), and the increase in forest stand density was significantly negatively associated with the decrease in anxiety, anger, and other negative emotions (6). The high-density canopy density induces a sense of gloom, and it limits visitors' immersion as people feel unsafe in the forest without sunlight (17). Similarly, the overgrowth of vegetation creates a sense of danger even while blocking the view of people and providing a sense of privacy (20, 26, 28). These findings confirm the classical environmental preference theory, such as Attention Restoration Theory (29), Preference Matrix (29), Stress Reduction Theory (30), Biophilia Hypothesis (31), Prospect-refuge Theory (32), and Supportive Environment Theory (33). These studies confirm that visual perception is one of the most important mediating variables of forest stand that influences visitors' willingness to visit and their evaluation. There is, however, no general agreement among researchers regarding the specifics behind the relationship between tree density and preference. The relationship between tree/vegetation density and preference is usually viewed as linear, some have described it as a power curve (21), while others found it to be quadratic (5). Due to these disagreements, it is difficult to apply our experiment results directly into forest parks improvement. So far, a lack of standardized measures has plagued most forest visual environment measurements. For example, landscape elements measured in 2D images cannot accurately reflect the 3D scene where multiple stand attributes interact, and it is not accurate to measure the vegetation permeability degree in a natural forest through a questionnaire survey of visual perception.

In addition, no consensus has been reached yet on how to present the environ-mental stimuli of the forest and test visual preference. The majority of visual preference studies are conducted in real forests environments since they offer a vivid and realistic view of the forest, however, the results may be affected by temperature, weather, and other perceptions (e.g., sound, smell) as well (12, 22, 34). Many other studies use 2D pictures and 2D video eye-tracking in the laboratory to simulate forest stimuli, which can record the eye movements and gaze of the participants when observing, and analyze the visual exploration mode and preference (35, 36). Although this laboratory method has the advantage of eliminating environmental interference, the feeling of immersion is weaker than the on-site experience, and the interaction with the forest environment is also limited by the absence of presence (37). Immersive Virtual Environment (IVE) device, as an emerging technology, provides a Virtual Reality (VR) experience that is compatible with the advantages of real (immersion and presence) and laboratory experiments (environmental variable controlled) to simulate the interaction between visitors and the forest environments (38–40). Recently, a lot of studies have verified the effectiveness of IVE in simulating real nature (3, 41), including the effect of a virtual forest environment on emotion regulation (42), stress recovery (43), and psychological restorative (44, 45).

Limited by the technical integration, rarely study on forest visual perception has combined the advantages of eye-tracking technology and IVE real-world simulation to discuss how vegetation permeability, which is formed by the interaction of multiple forest stand attributes, affect visitors' visual preference. The present study aims to add new insights to forest preference research, as well as to explore the application of VR eye-tracking technology in forest visual preference research. The specific objectives are the following:

1. Visual behavior characteristics of different vegetation permeability in VR forest exposure;
2. Psychological cognitive evaluation of different vegetation permeability in VR forest exposure;
3. The relationship between vegetation permeability, visual behavioral characteristics, and psychological cognitive evaluation.

## 2. Materials and methods

### 2.1. Study site and stimuli

**Figure 1:**
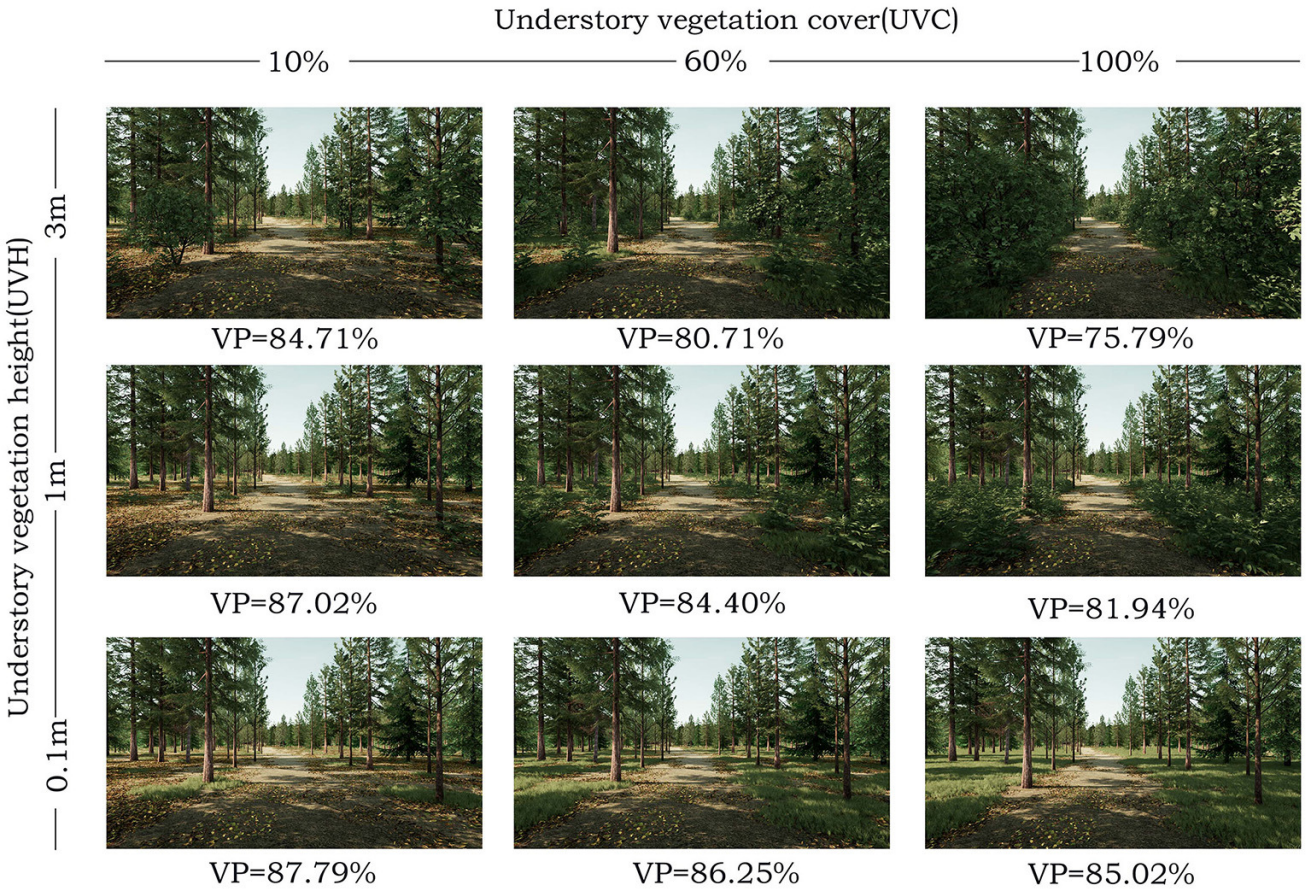
Research stimulus of coniferous forest in the virtual reality video display. VP, vegetation permeability.

**Figure 2:**
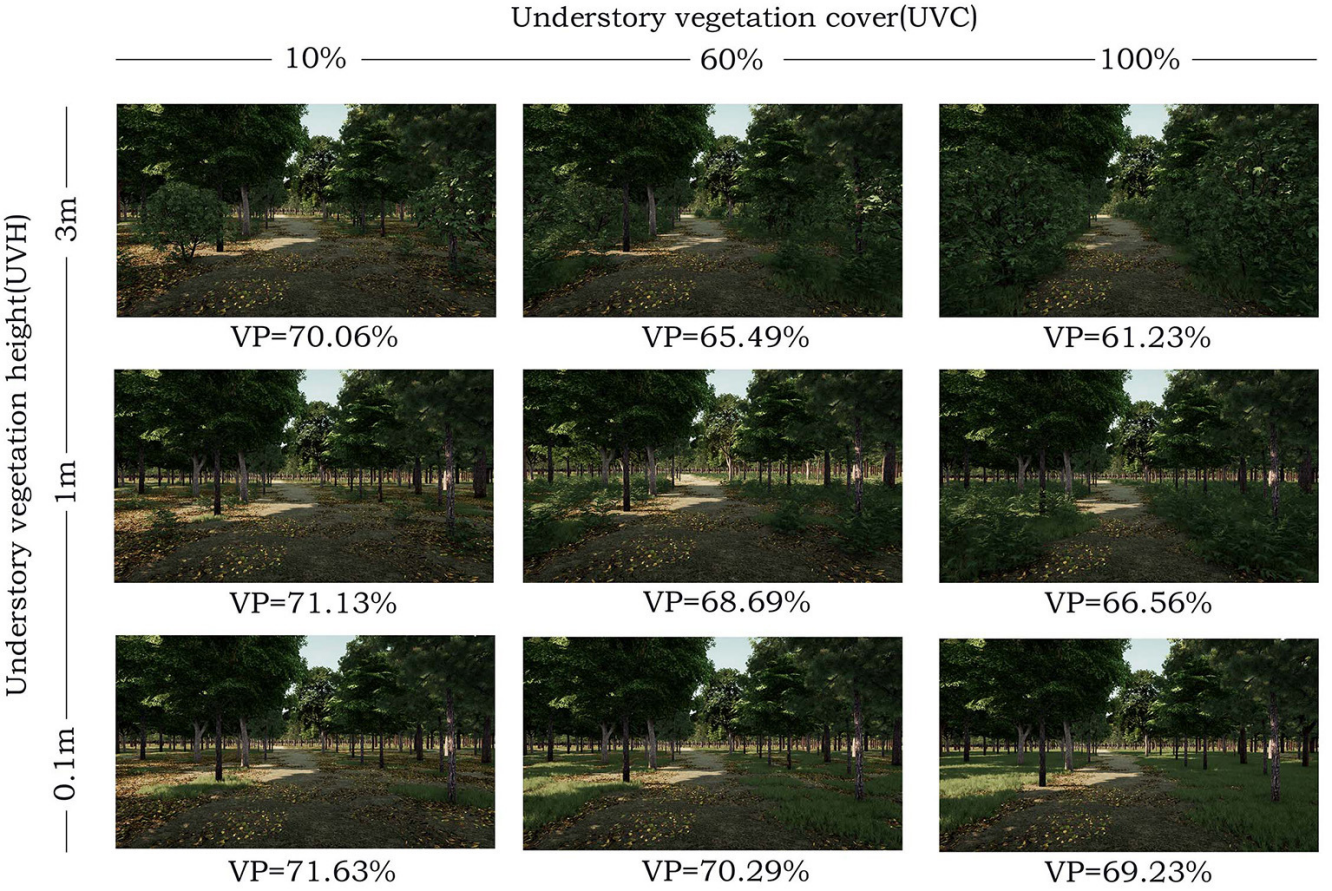
Research stimulus of broadleaved forest in virtual reality video display. VP, vegetation permeability.

Three subjective parameters include a location (longitude: 31.24628727, latitude: 120.58306217), time (12 am, 20 August 2019), and weather conditions (sunny) are set by virtual reality simulation software (Mars 2019, Sheencity Technology Ltd.) to define the virtual forest environmental attributes and regional plants, and a 10-hectare VR forest is created as experimental area. According to the classifying standards of the upper vegetation, stand attributes, and attribute levels in previous forest scene studies (6, 17, 27), the common coniferous and broadleaved forest scenes in southwest China are selected as stimuli. Each forest scene is set up with a stand density of 320 trees per hectare, this density value has been proven to be useful in facilitating visual access and promoting emotional and cognitive recovery (6, 17). The ground covering of forest scenes consists of under herbs, and undershrub, it includes two visual factors of UVC (level range: 0, 60, 100%) and UVH (level range: 0.1, 1, 3 m), as well as three different factor levels (Table 1; Figures 1, 2). These indicators are related to visual access and perception (20, 27, 46, 47). Since this study focuses on visual perception, other factors which may affect the validity of the experiment, such as water bodies, animals, recreational facilities, forest visitors, and traces of management, are excluded.

**Table 1:** Virtual reality forest stand attributes and meanings.

**Stand attribute**	**Level**	**Meanings**	**Dominant vegetation species**
FT	Coniferous forest	Low canopy density (59.19%)	*Metasequoia glyp-tostroboides, Taxodium Zhongshanshan, Pinus massoniana*
	Broadleaved forest	High canopy density (89.43%)	*Cinnamomum cam-phora, Eucalyptus robusta, Ficus virens*
UVC	10%	Almost bare ground	*Zoysia japonica Steud., Sambucusjavanica Blume, Ficus carica L*.
	60%	Most of the ground covered with vegetation	
	100%	Completely covered with ground vegetation	
UVH	0.1 m	High visibility and accessibility	
	1 m	High visibility and low accessibility	
	3 m	Low visibility and accessibility	

FT, forest type; UVC, understory vegetation cover; UVH, understory vegetation height.

**Figure 3:**
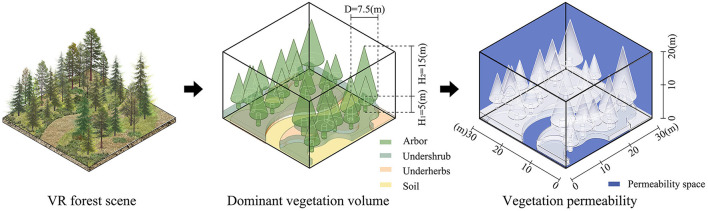
The experimental flow chart in vegetation permeability analysis.

Many studies quantify the landscape elements of 2D pictures to distinguish the differences between landscape elements in different scene types (20, 54). Based on these studies, the Boolean operation of SketchUp 2021 (Trimble Inc.) is used to calculate the vegetation permeability degree in 3D forest video. Firstly, it describes and calculates the volume with dominant vegetation species (i.e., tree and ground covering) in the VR forests scene; secondly, it subtracts the vegetation volume from the total volume; and finally, it calculates the index of vegetation permeability (Figure 3). The vegetation permeability of coniferous forests is 75.79–85.02% (Figure 1), while that of the broadleaved forest is 61.23–69.23% (Figure 2). The formula is as follows:


$$\begin{array}{l} \text{Vegetation permeability index}\\ =\left(\text{Total volume }-\text{Vegetation volume}\right)/\left(\text{Total volume}\right)\;\times 100\% \end{array}$$

**Figure 4:**
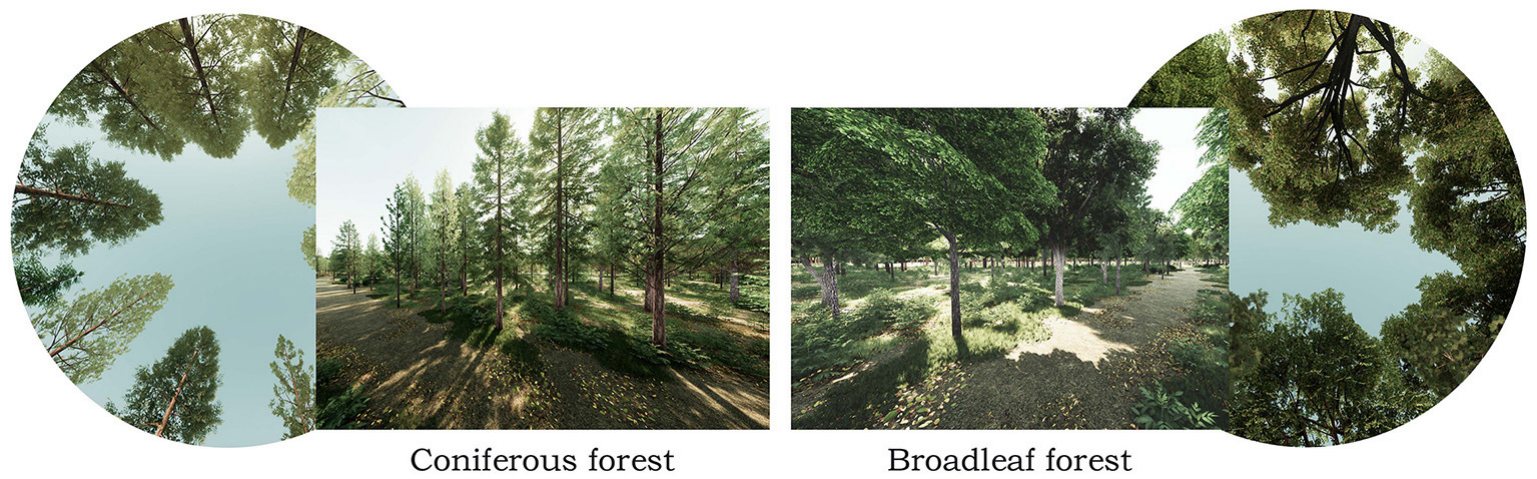
Schematic screenshots of VR video used as stimuli.

When compared to static stimuli, dynamic stimuli can generate more natural viewing behaviors (48), so we simulate the exposure of individuals while walking in the forest instead of sitting. As shown in Figure 4, we render 18 VR walking-simulated videos with different vegetation permeability but the same walking route and imitate the usual walking speed of adults (78–90 m/min).

### 2.2. Participants

To obtain a homogeneous sample to calibrate the method, we recruit 60 college students on campus social platforms, 14 of them are excluded due to the time conflict. 46 college students participate in the experiment, including 23 males and 23 females, aged between 19 and 27 (*M* = 23.33, SD = 1.96). Previous studies have shown that visitors aged between 18 and 35 are the main participants in forest park tourism (49), and college students are regarded as representative and convenience sample groups in visual stimuli research (50). All participants are Han people living in Southeast China, and no forestry major student participated for avoiding bias in preferences and perceptions of forests. Besides, none of them has any visual impairment or problem that may affect data collection. The results of the calculation with G-power version 3.1.9.2 highlight a study size of 36 each group [effect size *d* = 0.5, β = 0.05, and power (1–β) = 0.95].

### 2.3. Apparatus

A head-mounted virtual reality eye-tracker (Ergo VR, Kingfar Inc.), with head-mounted display (VIVE Pro, HTC Inc.), tracking frequency (120 Hz) and tracking range (≥120°), is employed to record eye movements. It is equipped with a test main-frame computer (Windows 10, Intel Core i9 CPU @ 3.6GHz, 64-bit, Nvidia GeForce RTX 2080Ti, 32 GB RAM) and two external lighthouses for tracking head position. By integrating virtual reality and eye-tracking into the head-mounted display. When the participants are freely viewing virtual scenes, their eyes and head move simultaneously. This makes researchers capture and record more natural visual behaviors than in experimentally controlled virtual environments (51, 52).

### 2.4. Experimental design

**Figure 5:**
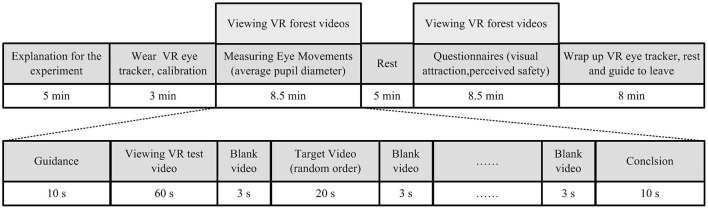
Flow diagram of the experimental procedure.

The study is performed in a quiet eye-tracking laboratory with room temperature constantly maintained at 25°C. Each experiment takes about 40 min, the details are as follows (Figure 5).

**Figure 6:**
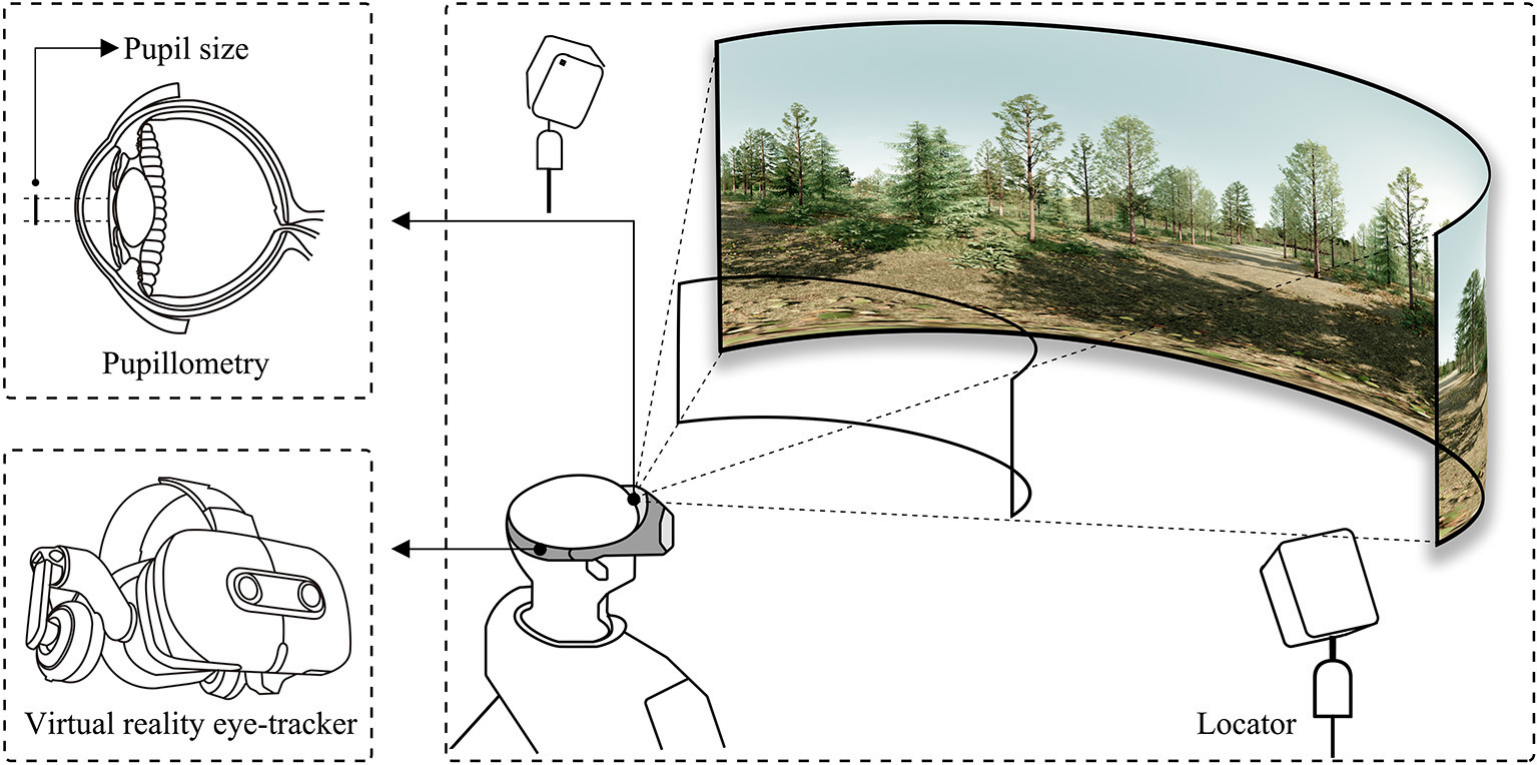
View schematic of the virtual reality eye-tracking experiment.

First of all, the participants are asked to wear a VR eye tracker of head-mounted which calibrates eye movements by repeated measurements of their eyes by infrared light reflection. After calibration, they turn to watch a 60 s test video to adapt to the VR viewing mode, during which researchers introduce the experiment procedures and precautions. Subsequently, 18 virtual reality videos are randomly presented on the display screen of the VR eye tracker, each target video is about 20 s, and the blank video was spaced 3 s. The participants are free to move their eyes and head to watch the videos during the experiment, and they could withdraw from the experiment at any time if they feel uncomfortable. This part of the experiment mainly obtains the visual physiological data of the participants when viewing different VR forest videos, especially the average pupil diameter (Figure 6). Average pupil diameter records the aver-age pupil size change of participants in each VR forest video. In previous studies, it has often been regarded as a predictor of emotional arousal and autonomic activation (53–55). In fact, a dilator stimulation or a constrictor inhibition can cause pupillary dilation. Its average size in standard light conditions is about 3 mm (56). And in dim light or darkness, the pupil can enlarge to an average size of about 7 mm (57). Cognitively driven changes are usually smaller and rarely exceed 0.5 mm (58).

Eye-tracking data can measure participants' visual physiological indicators but is unable to distinguish specific visual psychological characteristics. Therefore, after the eye-tracking experiment, the participants are asked to fill in demographic information (gender, age, educational level) and forest visit information (visit frequency, visit duration), and then display 18 virtual reality videos randomly again for evaluation. The relationship between the physical environment and visual preferences has been demonstrated in previous studies (59–61), and we select indicators of visual attractiveness and perceived safety to collect their visual psychological data. Between them, visual attraction is used to assess the perceived fascinating of visual environments (59), and perceived safety is often used to assess the perceived threat of the environment (8, 60). The primary sources of threat are social incivilities (e.g., physical or sexual assault), whereas in natural areas there are additional threats (e.g., predators, snakes, spiders). Each item is assessed by a 5-point Likert scale (“not at all” to “very much”).

### 2.5. Analysis and statistics

**Figure 7:**
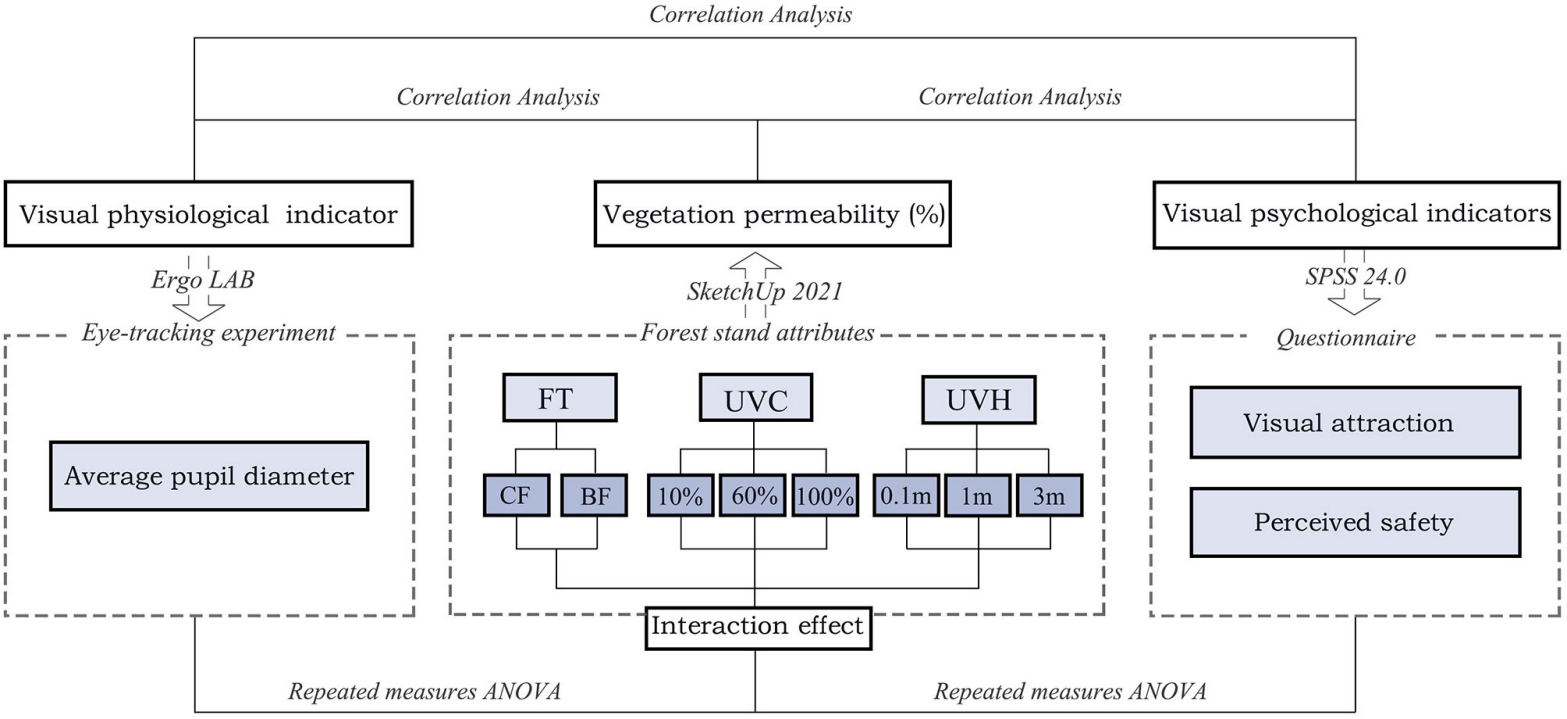
Research framework. FT, forest type; UVC, understory vegetation cover; UVH, understory vegetation height; BF, broadleaved forest; CF, coniferous forest.

We process the eye movement data through the eye-tracking software Ergo LAB (Kingfar Inc.), and the data analysis and psychological perception evaluation are con-ducted by SPSS 24.0 (IBM Inc.). First of all, we calculate the vegetation permeability of 18 VR videos with different stand attributes. Second, calculate the descriptive statistics including mean values and standard deviation for average pupil diameter, fascination, and perceived safety scores in different forest stand attributes. Third, assess the main effects of forest type (within-participants factor: coniferous forests and broadleaf forests), UVC levels (within-participants factor: 10, 60, 100%), and UVH levels (with-in-participants factor: 0.1, 1, 3 m), a three-factor mixed-design analysis of ANOVA is also conducted; besides, Box's M test is used to examine the homogeneity of co-variance assumption in all ANOVA models. For repeated measures with sphericity, Greenhouse Geisser (GG) correction is applied, and the effect size is calculated by partial Eta squared. Fourth, Tukey's HSD is performed to examine how forest type, UVC, and UVH affect average pupil diameter, the scores for fascination, and perceived safety separately between each level. Fifth, The Kendall and Spearman rank correlation is used to analyze correlations between vegetation permeability and participants' visual perception. Partial Eta squared is adopted to calculate the effect size (Figure 7). Significance is established at *p* < 0.05^*^, *p* < 0.01^**^.

## 3. Results

### 3.1. Effects of vegetation permeability on average pupil diameter of participants

The results of the ANOVA test reveal the main significant effects of forest type on participant's average pupil diameter [*F*_(1, 826)_ = 147.89, *p* < 0.01, ηp2 = 0.15], and indicate that viewing broadleaf forests has a higher average pupil diameter than viewing coniferous forests. Similarly, UVH has a significant main effect on average pupil diameter [*F*_(2, 825)_ = 3.57, *p* < 0.05, ηp2 = 0.01], and that viewing 100% UVH has higher average pupil diameter than viewing 10% (*p* < 0.05; Tables 2, 3).

**Table 2:** Descriptive statistics for average pupil diameter, the scores of visual attraction and perceived safety.

**Stand attribute (VF)**	**Average pupil diameter**	**Visual attraction**	**Perceived safety**
**FT**			
Coniferous forests (83.74 ± 3.74)	3.18 ± 0.47	3.20 ± 0.93	3.17 ± 0.99
Broadleaf forests (68.31 ± 3.36)	3.63 ± 0.58	3.22 ± 0.93	2.97 ± 1.04
**UVC**			
10% (78.81 ± 8.50)	3.36 ± 0.56	3.13 ± 0.89	3.22 ± 0.97
60% (75.97 ± 8.88)	3.40 ± 0.57	3.30 ± 0.92	3.07 ± 0.98
100% (73.30 ± 9.23)	3.45 ± 0.58	3.20 ± 0.97	2.92 ± 1.08
**UVH**			
0.1 m (78.37 ± 8.82)	3.35 ± 0.58	3.22 ± 0.90	3.39 ± 0.91
1 m (76.62 ± 8.85)	3.39 ± 0.55	3.30 ± 0.91	3.19 ± 0.93
2 m (73.08 ± 9.00)	3.47 ± 0.58	3.12 ± 0.97	2.62 ± 1.06

Data are shown as Mean ± SD. VP, vegetation permeability (%); FT, forest type; UVC, understory vegetation cover; UVH, understory vegetation height.

**Table 3:** ANOVA analysis for average pupil diameter, the scores of visual attraction, and perceived safety.

	**Average pupil diameter**				**Visual attraction**				**Perceived safety**			

	**df**	***F***	η**p**^2^	***p***	**df**	***F***	η**p**^2^	***p***	**df**	***F***	η**p**^2^	***p***
FT	1.826	147.89	0.15	<0.001	1.826	0.11	<0.001	0.737	1.826	8.06	0.01	0.005
UVC	2.825	1.90	0.01	0.150	2.825	2.26	0.01	0.105	2.825	6.07	0.02	0.002
10 vs. 60%				0.701				0.089				0.183
10 vs. 100%				0128				0.688				0.002
60 vs. 100%				0.492				0.405				0.198
UVH	2.825	3.57	0.01	0.029	2.825	2.74	0.01	0.065	2.825	46.96	0.10	<0.001
O.1 vs. 1 m				0.605				0.543				0.047
O.1 vs. 3 m				0.023				0.405				<0.001
1 vs. 3 m				0.213				0.051				<0.001

FT, forest type; UVC, understory vegetation cover; UVH, understory vegetation height.

**Figure 8:**
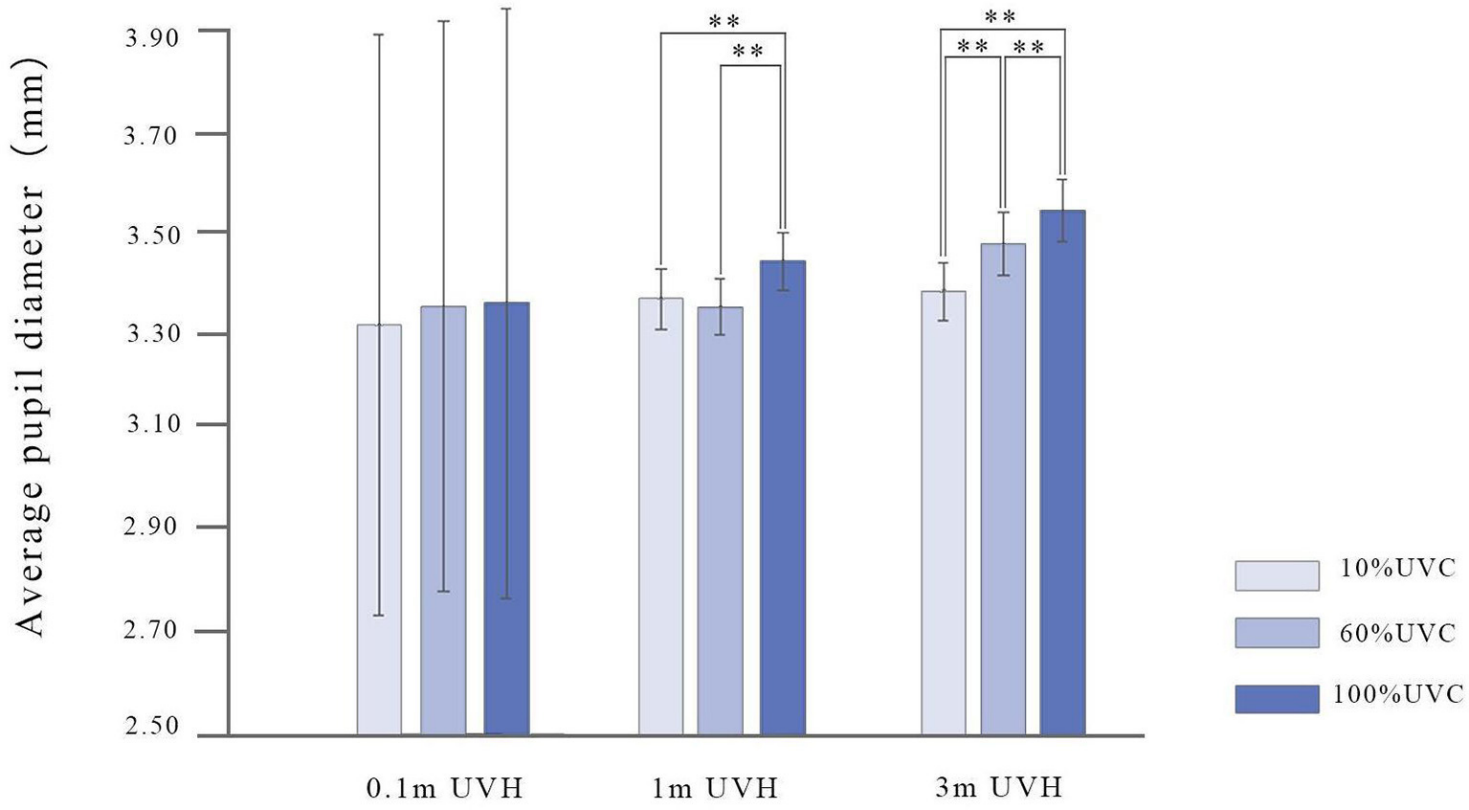
The interaction of UVC and UVH on average pupil diameter. UVC, understory vegetation cover; UVH, understory vegetation height. **p < 0.01.

**Figure 9:**
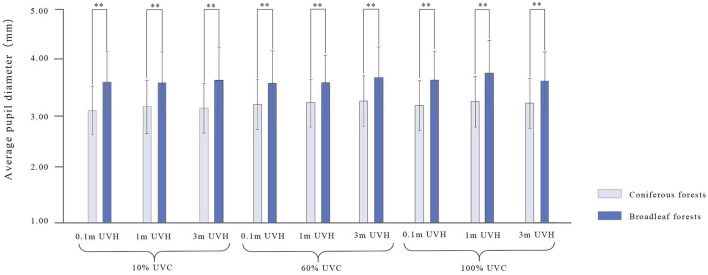
The interaction difference of UVC and UVH on average pupil diameter in two forest types. UVC, understory vegetation cover; UVH, understory vegetation height. **p < 0.01.

Significant interaction effect has been found between UVC and UVH [*F*_(4, 540)_ = 5.51, *p* < 0.01, ηp2 = 0.04; Figure 8]. Tukey's HSD *post-hoc* analysis indicates that 100% UVC with 1 m UVH on average pupil diameter is higher than 10% (*p* < 0.01) and 60% at the same height (*p* < 0.01), and the average pupil diameter of different UVC with 3 m UVH is significantly different, namely, 10 vs. 60% (*p* < 0.01), 10 vs. 100% (*p* < 0.01), 60 vs. 100% (*p* < 0.01; Figure 8). Additionally, when both forest types are considered, a significant interaction difference has been found between UVC and UVH [*F*_(2, 540)_ = 2.77, *p* < 0.03, ηp2 = 0.02]; Tukey's HSD *post-hoc* analysis indicates that, for the participants, the average pupil diameter of viewing nine coniferous forest scenes is significantly smaller than viewing broadleaved forest (all *p* < 0.01) with the same UVC and UVH indicators (Figure 9).

### 3.2. Effects of vegetation permeability on visual attraction

**Figure 10:**
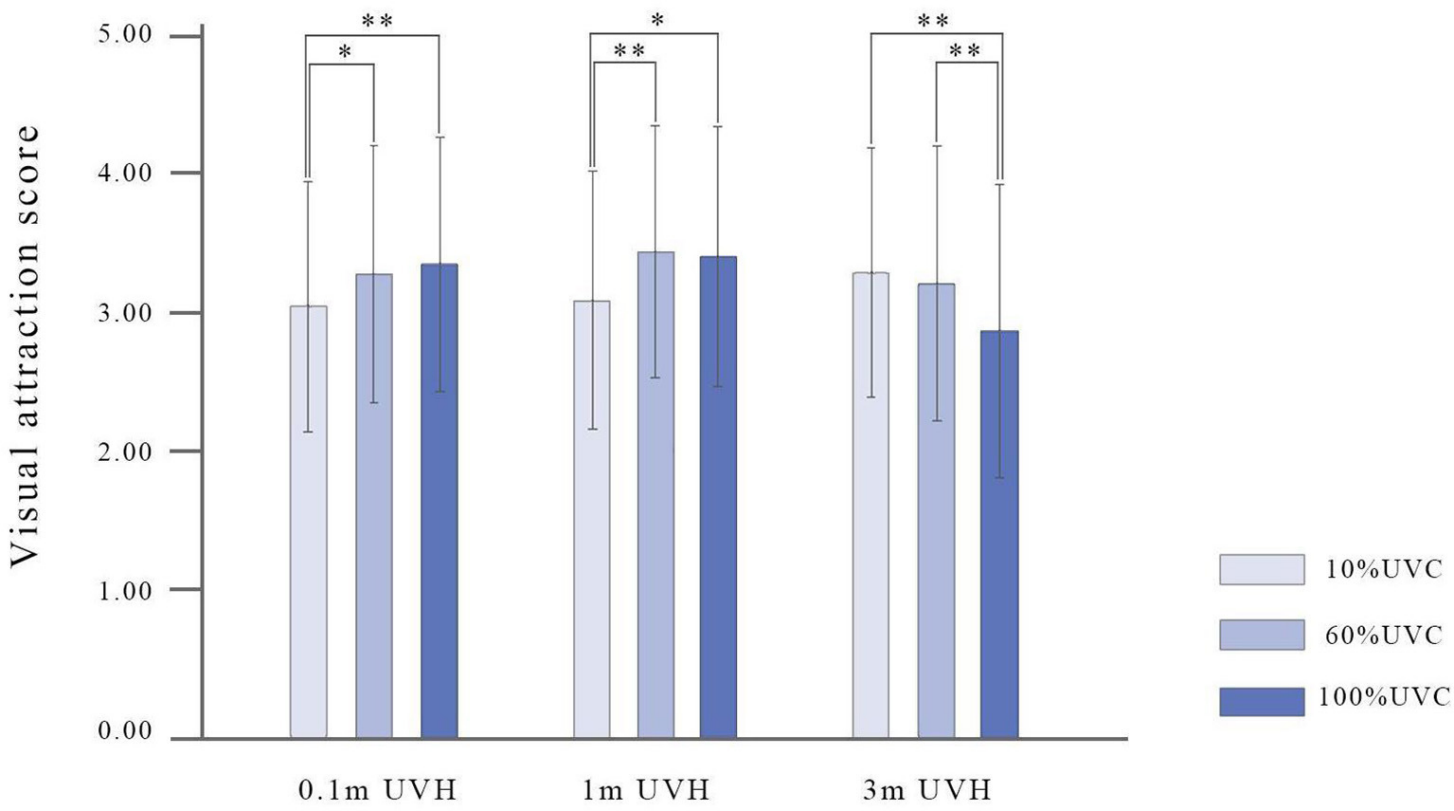
The interaction of UVC and UVH on visual attraction. UVC, understory vegetation cover; UVH, understory vegetation height. *p < 0.05; **p < 0.01.

The results of the ANOVA test indicate that no significant main effect of the three forest stand attributes (FT, UVC, and UVH) has been found on participant's visual at-traction (Tables 2, 3), but significant interaction effect exits between UVC and UVH [*F*_(4, 540)_ = 6.82, *p* < 0.01, ηp2 = 0.05; Figure 10]. Tukey's HSD *post-hoc* analysis shows that the effect of 10% UVC with 0.1 m UVH on visual attraction is significantly lower than 60% (*p* < 0.05) and 100% (*p* < 0.01) at the same height, the effect of 10% UVC with 1 m UVH on visual attraction is significantly lower than 60% (*p* < 0.01) and 100% (*p* < 0.05), and the effect of 100% UVC with 3 m UVH on visual attraction is significantly lower than 10% (*p* < 0.01) and 60% (*p* < 0.01) (Figure 10).

### 3.3. Effects of vegetation permeability on perceived safety

The results of the ANOVA test reveal the main significant effects of forest type on participant's perceived safety [*F*_(1, 826)_ = 8.06, *p* < 0.01, ηp2 = 0.01], and indicate that viewing coniferous forests has higher perceived safety than viewing broadleaved forests. Similarly, UVC has significant main effect on perceived safety [*F*_(2, 825)_ = 60.71, *p* < 0.01, ηp2 = 0.02], and the results show that viewing 10% UVC has higher perceived safety than 100% (*p* < 0.05). In addition, UVH also has a significant main effect on perceived safety [*F*_(2, 825)_ = 46.96, *p* < 0.01, ηp2 = 0.10], specifically, viewing 0.1 m UVH has higher perceived safety than viewing 1 m (*p* < 0.05) and 3 m (*p* < 0.01), and viewing 1 m has higher perceived safety than 3 m (*p* < 0.01) (Tables 2, 3).

**Figure 11:**
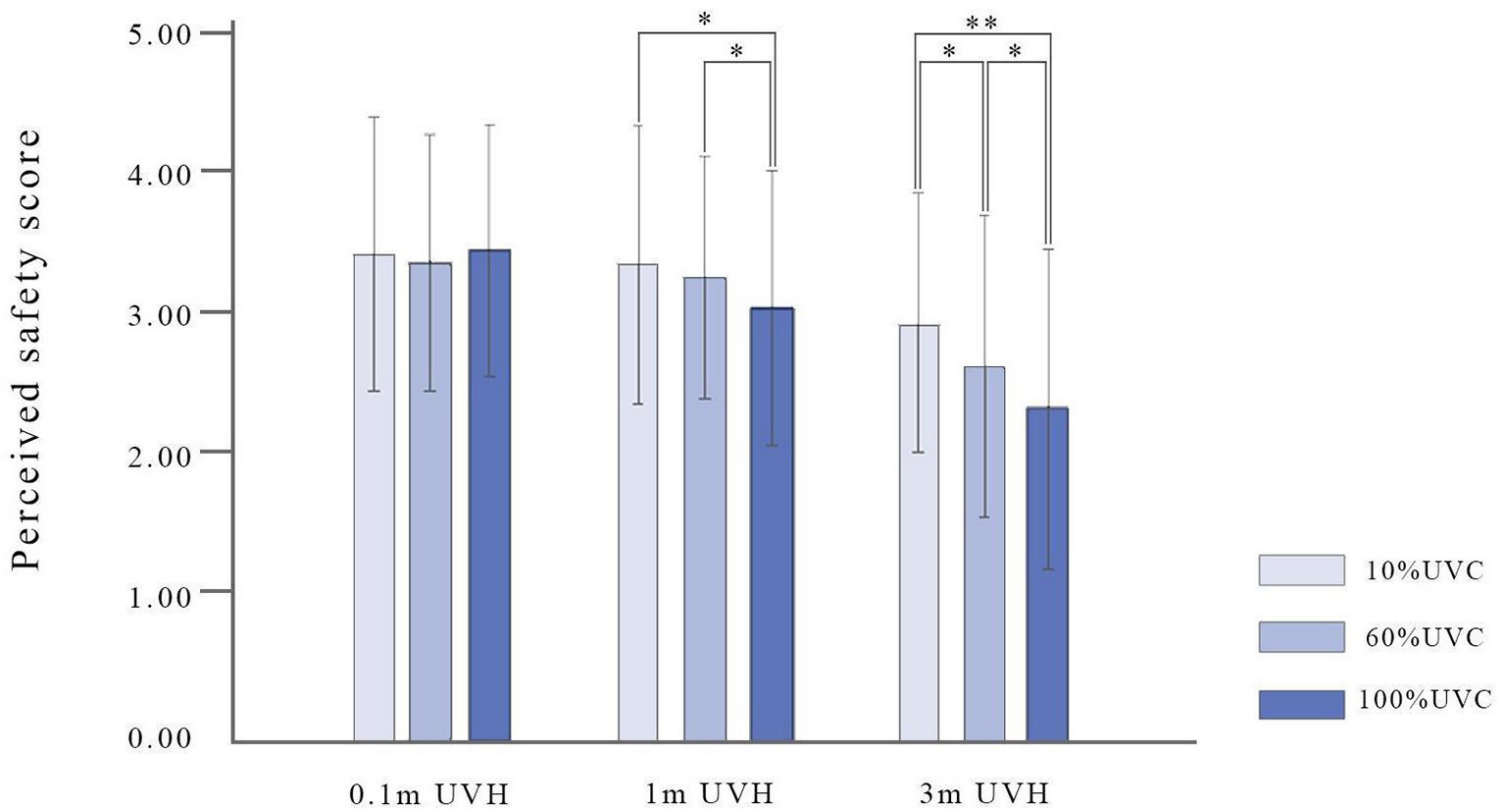
The interaction of UVC and UVH on perceived safety. UVC, understory vegetation cover; UVH, understory vegetation height. *p < 0.05; **p < 0.01.

Significant interaction effect has been found between UVC and UVH [*F*_(4, 540)_ = 3.37, *p* < 0.01, ηp2 = 0.02; Figure 11]. Tukey's HSD *post-hoc* analysis indicates that 100% UVC with 1 m UVH on perceived safety is significantly lower than 10% (*p* < 0.05) and 60% (*p* < 0.05) at the same height, and the perceived safety of different UVC at 3 m UVH is significantly different, namely, 10 vs. 60% (*p* < 0.05), 10 vs. 100% (*p* < 0.01), 60 vs. 100% (*p* < 0.05) (Figure 11).

### 3.4. Correlation between vegetation permeability and participant's visual perception

Table 4 shows the correlations between vegetation permeability and some participants' visual perceptions. The vegetation permeability is strongly negatively related to average pupil diameter (*r* = −0.40, *p* < 0.01), and strongly positively related to perceived safety (*r* = 0.22, *p* < 0.01). In contrast, the forest type is strongly positively related to average pupil diameter (*r* = 0.31, *p* < 0.01) and strongly negatively related to perceived safety (*r* = −0.09, *p* < 0.01). UVC level is strongly negatively related to perceived safety (*r* = −0.10, *p* < 0.01). UVH level is strongly positively related to average pupil diameter (*r* = 0.07, *p* < 0.01) and strongly negatively related to perceived safety (*r* = −0.27, *p* < 0.01).

**Table 4:** Correlation test between forest stand attributes and participant's visual perception.

	**Average pupil diameter**	**Visual attraction**	**Perceived safety**
Vegetation permeability	−0.40^**^	−0.02	0.22^**^
FT	0.31^**^	0.01	−0.09^**^
UVC	0.05	0.03	−0.10^**^
UVH	0.07^**^	−0.04	−0.27^**^
Average pupil diameter		−0.01	−0.08^**^
Visual attraction			0.16^**^

FT, forest type; UVC, understory vegetation cover; UVH, understory vegetation height. ^**^p < 0.01.

Besides, a Spearman rank order correlation analysis has also been performed to test the association between eye movement indicators and psychological perception evaluation indicators of participants. Table 4 shows the significant correlations be-tween perceived safety and other indicators, that is, perceived safety is strongly negatively related to average pupil diameter (*r* = −0.08, *p* < 0.01) and strongly positively related to a visual attraction (*r* = 0.16, *p* < 0.01).

## 4. Discussion and limitations

### 4.1. Discussion

In general, the results of VR eye-tracking and questionnaires have supplemented the thematic literature on forest stand attributes and perceived preferences (6, 10, 17, 20, 59, 62). Through VR simulation, vegetation permeability calculation, and immersive viewing experiments, the study draws out a principal finding that when viewing VR forests with different stand attributes,3D vegetation permeability [which is formed by the interaction effect of canopy density (FT) and understory vegetation density (UVC × UVH)] significantly affects participants' visual perception of visual physiological and visual psychological responses.

In addition, the study explores the effect of different vegetation permeability on the visual physiology of the participants, and the results indicate that pupil size is significantly correlated to the permeability of the forest environment, specifically, the lower the permeability of the forest environment, the larger the average pupil diameter. The study also confirms that the average pupil diameter, which reflects the visual physiological stress level, becomes larger when the participants are viewing forest scenes with high canopy density (e.g., broadleaved forest), low visibility, and ground accessibility than viewing forest scenes of low canopy density (e.g., coniferous forest), high visibility and ground accessibility. Two explanations can be put forward for these results. On the one hand, the illumination of closed forest stand attributes is at a low level and requires participants to dilate their pupils to adapt to the dark environment. On the other hand, the atmosphere of the closed forest stand attribute seems potentially threatening, and it may arouse the visual vigilance of the participants to identify possible threats. Previous studies in the restorative environment also confirm that pupil dilation is associated with arousal (53, 54).

Another noteworthy finding is about vegetation permeability and psychological perception evaluation in virtual forest environments. In terms of visual attractiveness, the understory density which is qualified by the interaction of UVC and UVH affects visual attractiveness significantly (Table 2), but the stand attribute variables (FT, UVC, and UVH) are not significantly correlated to visual attractiveness variables in statistics (Table 3). This difference may be caused by a curvilinear relationship between two variable groups. Visual attractiveness improves with the increase of understory density until a certain threshold is reached, after which the relationship gradually weakens (Figure 5). Thus, preference for visual attractiveness is correlated with moderate understory density, namely, it favors high UVH in low UVC and low UVH in high UVC. These results are in line with many previous works, such as the visitor's preference for forest scenarios with a certain complexity (27, 63, 64), and the inverted-U shape relationship between tree cover density and stress recovery (20, 65).

The results of the study on perceived security indicate that the less vegetation permeability, the lower the visual security score. That is, both the decrease of sky visibility due to the increase of canopy density and the increase of understory density formed by the interaction of UVC and UVH will lead to a decrease in the perceived safety of participants. The findings corroborate previous studies of landscape environment preference. The public prefers a natural environment with a wide view since the openness of the surroundings makes it possible for them to check up (the sky) or down (the ground), which brings them a sense of security (21, 47, 56, 61).

Another important finding is that the average pupil diameter and perceived safe-ty are significantly negatively correlated, which confirms the feasibility of pupil size in predicting visual safety in forest environments. In previous eye-tracking experiments, pupil size is usually used to assess visual recovery in natural or artificial environments (36, 54). In psychological perception evaluation indicators, the study finds a significant positive correlation between visual attraction and perceived safety, which coincides with the earlier findings. Previous evaluations of landscape environment preference have shown that the perceived safety of the natural environment is a prerequisite for attracting visitors and arousing aesthetic pleasure, it is an adaptive mechanism for humans to avoid danger in the evolutionary process (66, 67). Additionally, no correlation has been found between average pupil diameter and visual attraction, which may be attributed to the fact that pupil size cannot be used as a dimension to assess visual attraction.

### 4.2. Limitations

In the present study, we employ VR eye-tracking technology to explore visual perception in an immersive 3D forest environment. When compared with the experiments adopted in real forests by wearing eye-tracking glasses or viewing forest photos with eye-tracking devices, VR eye-tracking technology has the advantage of simulating and collecting the continuous gaze behavior of participants when they are walking in the forest and eliminating occasional environmental disturbances (such as birds, wind, and light changes). The study has verified the effectiveness of VR eye-tracking technology in VR forest environment from the perspective of visual perception. It is an extension to the previous VR forest environment researches in emotional improvement, stress restorative, and psychological restorative. However, some limitations of experimental design and data analysis need to be considered. Firstly, due to space limitation, vegetation permeability mentioned in this study only includes the interaction effects of three forest stand attributes (FT, UVC, and UVH) and other forest stand attributes, such as stand density, stand area, and tree height (10, 11, 17), will be explored in the future; secondly, it is time-consuming to manually analyze the 3D vegetation permeability data of 18 VR forest panoramic videos. It is necessary to improve analysis efficiency in the future to reduce the workload of researchers; furthermore, the participants are college students, and although scholars in previous studies have indicated that college students are representative samples (49, 50), the demographic characteristics of the participants may influence their perceived preference for the forest stand structure (17, 60). For the participants of the present study, the influence of their age, gender, educational back-ground, and major has not been discussed. Future studies should consider these demo-graphic characteristics.

## 5. Conclusions

The present study focuses on vegetation permeability which is formed by FT, UVC, and UVH), and how they affect participants' visual perception when viewing a VR forest scene. The results show that vegetation permeability formed by the interactive effect of canopy density (FT) and understory density (UVC × UVH) significantly affects participants' visual perception. In terms of visual physiology characteristics, the pupil size of the participants is significantly correlated to vegetation permeability when they are viewing the VR forest, specifically, the lower the permeability of the forest environment, the larger the average pupil diameter.

In terms of visual psychological characteristics, the understory vegetation density formed by the interaction of UVC and UVH has a significant impact on visual attractiveness and perceived safety, and it is significantly negatively correlated with perceived safety. In addition, the study also finds a significant negative correlation between average pupil diameter and perceived safety, which confirms the usefulness of pupil size in assessing perception safety in a forest environment. These results supplement existing literature on relationships in forest restorative environments.

For the research method, the study pioneers the use of VR eye tracking to explore the visual perception of vegetation permeability and confirms that this technology could be successfully used to collect visual physiology data by viewing forest scenes. Furthermore, the VR forest simulation provides an opportunity to systematically manipulate environmental stimuli for strict experiments which are designed to evaluate the impact of various environmental variables on individual visual physiology and psychology.

The results are also beneficial to forest park practice. When designing or upgrading forest parks, it could be crucial to consider the vegetation permeability, which is the interaction effect of different forest stand attributes on the visual perception of visitors. Last but not the least, the VR eye-tracking and visual assessment methods employed in this study could advance relative urban public space research, as well as provide potential benefits to evaluate the continuous experience of on-site visual perception in other public spaces (e.g., waterfronts, community parks, and street green spaces).

## Data availability statement

The raw data supporting the conclusions of this article will be made available by the authors, without undue reservation.

## Ethics statement

The studies involving human participants were reviewed and approved by Ethics Committee of Suzhou University of Science and Technology. The patients/participants provided their written informed consent to participate in this study.

## Author contributions

CL: conceptualization, methodology, and writing. CD: project administration and funding acquisition. TT: software and visualization. SG: data curation, visualization, validation, and software. All authors contributed to the article and approved the submitted version.
