# Supplementary material for: An eye-tracking study on visual perception of vegetation permeability in virtual reality forest exposure
Source: Front Public Health. 2023 Jan 24;11:1089423. doi: 10.3389/fpubh.2023.1089423 (PMC9902884; doi:10.3389/fpubh.2023.1089423)
Supplement: Supplementary file 1 [file Data_Sheet_1.docx]

Supplementary Material

An eye-tracking study on visual perception of vegetation permeability in virtual reality forest exposure

**Chang Li ^1*^ , Chunlan Du ^2*^ , Shutong Ge ^1^ and Tong Tong ^1^**

*** Correspondence:** Corresponding Author: lichang@usts.edu.cn

# QUESTIONNAIRE (AFTER the VR video)

Section A – Socio-demographics

① Male ____Female____

② Age_________________

③ Ethnic_________________

④ Education Level_________________

⑤ Major_________________

⑥ Birthplace_______________

⑦ Frequency of Forest Visit /Year _______________

Section B- Measurement scale of visual psychological

We will now show you some VR videos that display different types of forest. We ask you to carefully watch the videos and choice the answer related to your psychological mind state. Please answer naturally and as honestly as possible.

(1) what extent do you perceive the scene as visual attraction? _______________

① Not at all

② A little bit

③ Somewhat

④ A lot

⑤ Very much

(2) To what extent do you perceive the scene as safety? _______________

① Not at all

② A little bit

③ Somewhat

④ A lot

⑤ Very much
